# Survivin drives renal cell carcinoma tumorigenesis via proliferative and metabolic adaptation

**DOI:** 10.1242/dmm.052706

**Published:** 2026-08-25

**Authors:** Shivani Tuli, Gabrielle Inserra, Yamato Murakami, Cais Vo, Sarvesh Senguttavan, Yongho Bae, Dave Gau

**Affiliations:** ^1^ Department of Bioengineering, University of Pittsburgh, Pittsburgh, PA 15213, USA; ^2^ Department of Pathology and Anatomical Sciences, University at Buffalo, Buffalo, NY 14260, USA; ^3^ Department of Biology, University of Pittsburgh, Pittsburgh, PA 15213, USA; ^4^ Department of Pathology, University of Pittsburgh, Pittsburgh, PA 15213, USA

**Keywords:** Survivin, Renal cell carcinoma, Proliferation, Migration, Mitochondria

## Abstract

Renal cell carcinoma (RCC) is a heterogeneous malignancy for which clear cell RCC (ccRCC) represents the most common and clinically aggressive subtype. Survivin (encoded by *BIRC5*), an inhibitor of apoptosis and key regulator of mitosis, is frequently overexpressed in RCC and associated with poor prognosis, yet its broader role in kidney cancer biology remains poorly defined. By analyzing transcriptomic data from The Cancer Genome Atlas-kidney renal clear cell carcinoma (TCGA-KIRC) cohort, we found that advanced-stage ccRCC exhibits widespread dysregulation of cell-cycle pathways, with 15,160 genes upregulated and 479 genes downregulated in stage IV compared to stage I tumors. To define survivin's functional contribution, we performed loss-of-function and pharmacologic inhibition studies in murine RENCA and human 786-O RCC cell lines. Survivin knockdown or treatment with the small-molecule inhibitor YM155 reduced proliferation, S-phase entry and cyclin D1 expression, and impaired collective (wound-healing), single-cell and transwell migration. Unexpectedly, survivin depletion increased mitochondrial content while lowering oxygen consumption, indicating accumulation of dysfunctional mitochondria. Together, these findings identify survivin as a node between cell-cycle progression and mitochondria in RCC.

## Teaser

**Summary:** Survivin drives tumor growth and migration via proliferative and metabolic adaptation and could be a therapeutic target for renal cell carcinoma.

## INTRODUCTION

Renal cell carcinoma (RCC) accounts for ∼85% of all primary renal neoplasms and is the ninth most common cancer in the United States, with an estimated 80,980 new cases and 15,160 deaths projected in 2026 (Siegel et al., 2025). Arising from renal tubular epithelial cells, RCC is a heterogeneous disease, with several histologically and molecularly distinct subtypes. Clear cell RCC (ccRCC) is the most common subtype, accounting for over 75% of RCC cases, followed by papillary RCC (15-20%) and chromophobe RCC (5%). Among these subtypes, ccRCC has the worst clinical outcomes, with 30% of patients presenting with local recurrence or distant metastases after initial treatment. Outcomes depend heavily on stage at diagnosis: the overall 5-year survival rate is 78.1%, but in metastatic disease median survival falls to ∼13 months and 5-year survival to under 10% (Leibovich et al., 2003).

The dual role of survivin (encoded by *BIRC5*) as an inhibitor of apoptosis and a regulator of mitosis is well documented. It suppresses apoptosis by interacting with caspases-3, -7 and -9 and stabilizes mitochondrial integrity through its association with X-linked IAP (XIAP) and other apoptotic regulators (Dohi et al., 2004; Jaiswal et al., 2015). Additionally, survivin functions as a component of the chromosomal passenger complex, in which it ensures proper chromosome alignment and segregation during mitosis by regulating microtubule dynamics (Knauer et al., 2006; Vader et al., 2006). However, emerging evidence suggests that survivin's impact extends beyond these canonical roles to include regulation of early cell-cycle progression, mitochondrial function and metabolic reprogramming, processes that are critical for tumor progression but remain poorly understood in kidney cancer (Li et al., 2021).

Mitochondria are central regulators of energy metabolism, apoptosis and cellular homeostasis. In cancer cells, mitochondrial dynamics, specifically the processes of fission and fusion, are often dysregulated to support rapid proliferation and resistance to stress. Survivin has been implicated in modulating mitochondrial function by localizing to mitochondria and interacting with chaperones such as heat shock protein 90 (Hsp90) (Fortugno et al., 2003). These interactions stabilize mitochondrial integrity and prevent the release of pro-apoptotic factors such as cytochrome C, thereby promoting cell survival under stress conditions (Jaiswal et al., 2015; Hennigs et al., 2020). Mechanistically, survivin inhibits mitophagy by blocking parkin recruitment to dysfunctional mitochondria, leading to the accumulation of respiratory-defective mitochondria (Townley and Wheatley, 2020). Mitophagy inhibition forces cancer cells to rely on glycolysis for energy production, a hallmark of metabolic reprogramming known as the Warburg effect.

Despite growing recognition of survivin's role in mitochondrial function and metabolism, consequences of its knockdown in kidney cancer remain poorly understood. Furthermore, most studies have relied on standard cancer cell lines (e.g. HeLa or MCF-7), limiting their relevance to kidney cancer, a malignancy with unique metabolic dependencies. Addressing this gap is essential for advancing our understanding of survivin's multifaceted roles in kidney cancer biology. In this study, we investigated survivin's multifaceted role in renal carcinogenesis using a murine kidney cancer model system. Through survivin knockdown experiments, we uncovered its critical involvement in mitochondrial dynamics and energy homeostasis, interconnected processes essential for cancer cell proliferation and migration. Survivin depletion induces systemic dysregulation across these pathways, impairing mitochondrial architecture, metabolic plasticity and structural integrity. These disruptions culminate in suppressed proliferative capacity and compromised metastatic behavior.

Our work resolves a knowledge gap by establishing survivin as a central regulator of mitochondrial–structural coupling in kidney cancer. The coordinated collapse of bioenergetic and cytoskeletal networks upon survivin loss reveals an exploitable metabolic–structural vulnerability. This integrative perspective repositions survivin as a linchpin oncogenic hub, where its targeting could simultaneously disrupt tumor bioenergetics, growth signaling and motility machinery. By bridging mitochondrial function with cellular architecture, these findings provide a framework for developing combination therapies that exploit survivin's dual roles in renal carcinoma progression.

## RESULTS

### Survivin overexpression predicts aggressive RCC and poor patient survival

**Fig. 1.**
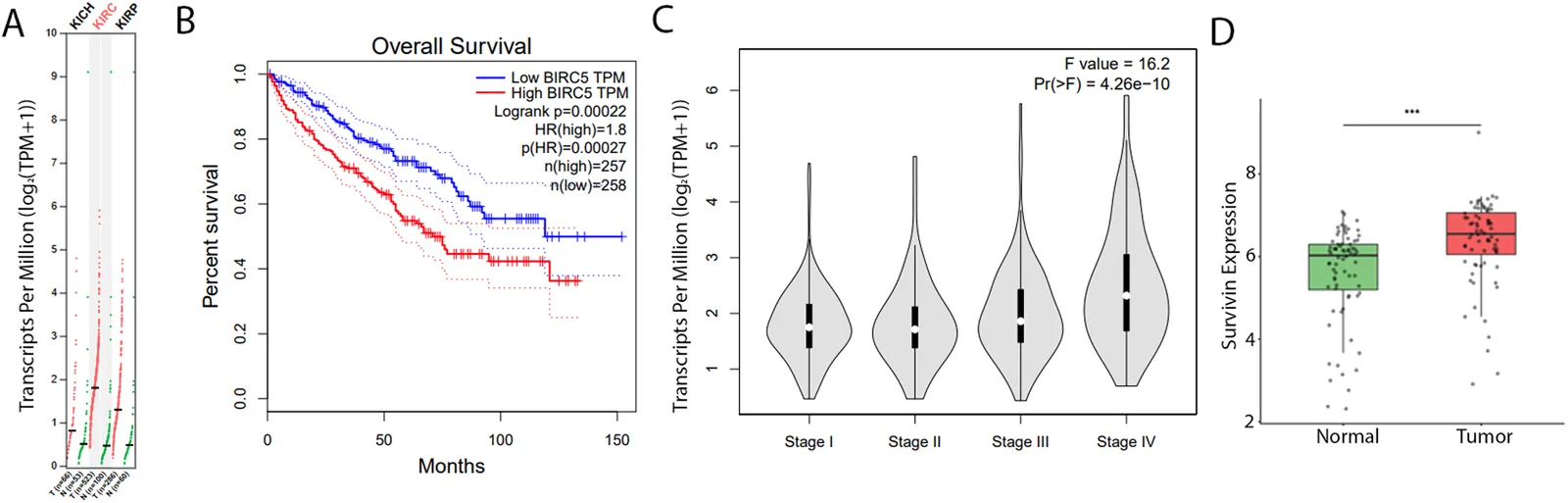
**Survivin is transcriptionally upregulated in the vast majority of human renal cell carcinomas (RCCs).** (A) Relative *BIRC5* mRNA expression between tumors (T) of different histological subtypes of kidney cancer and normal kidney tissue (N). Patient sample numbers for different subtypes: chromophobe (T=66, N=53), clear cell (T=523, N=100) and papillary (T=286, N=60). (B) Kaplan–Meier survival curve of The Cancer Genome Atlas (TGCA)-kidney renal clear cell carcinoma (KIRC) dataset separated by median expression of survivin. (C) Survivin expression plotted by different stages of clear cell RCC (ccRCC). Data were plotted on the Gene Expression Profiling Interactive Analyses (GEPIA) platform that uses transcriptome data from the TCGA and Genotype Tissue Expression (GTEx) databases. Pr(>F), *P*-value for *F* test. (D) Independent validation in the GSE53757 ccRCC microarray cohort (Affymetrix GPL570; *n*=72 tumor, *n*=72 matched normal kidney), showing higher *BIRC5* expression in tumor (red) versus normal tissue (green) (Wilcoxon ***P=9.96×10^−67^).

Survivin is aberrantly overexpressed in all three key histological subtypes of human RCC (with the most significant increase of expression in ccRCC) (Fig. 1A). Given that ccRCC is the most abundant form of kidney cancer, we focused on this subtype and found that high expression of survivin (segregated by median expression) showed worse patient prognosis (hazard ratio, 1.8; log-rank *P*=0.00027; Fig. 1B), underscoring its clinical relevance as a biomarker for poor outcomes. High survivin levels in patients were strongly associated with advanced disease features such as higher-stage tumor (Fig. 1C). This finding is comparable to those of a previous study showing that RCC patient cohorts with high survivin expression had significantly shorter overall survival than those with low expression (5-year cancer-specific survival ∼43% versus ∼87%, *P*<0.001), and, in addition, multivariate Cox regression confirmed survivin as an independent prognostic factor (after controlling for stage and grade, high tumoral survivin conferred a greater hazard of death with hazard ratio >2, *P*<0.01) (Parker et al., 2006). To independently validate the transcriptomic findings from The Cancer Genome Atlas (TGCA)-kidney renal clear cell carcinoma (KIRC), we examined *BIRC5* expression in the GSE53757 ccRCC microarray cohort (Affymetrix GPL570; *n*=72 tumor and *n*=72 matched normal kidney), which is independent of TCGA. In this independent cohort, *BIRC5* expression was significantly higher in tumor versus matched normal kidney tissue (Wilcoxon *P*=9.96×10^−7^; Fig. 1D), confirming the tumor-associated upregulation of survivin in a second, non-TCGA cohort. These data suggest that survivin expression correlates with RCC aggressiveness and poor patient prognosis, supporting its utility as a biomarker for aggressive disease.

### Transcriptomic analysis of TCGA-KIRC reveals stage-specific dysregulation of cell-cycle pathways

**Fig. 2.**
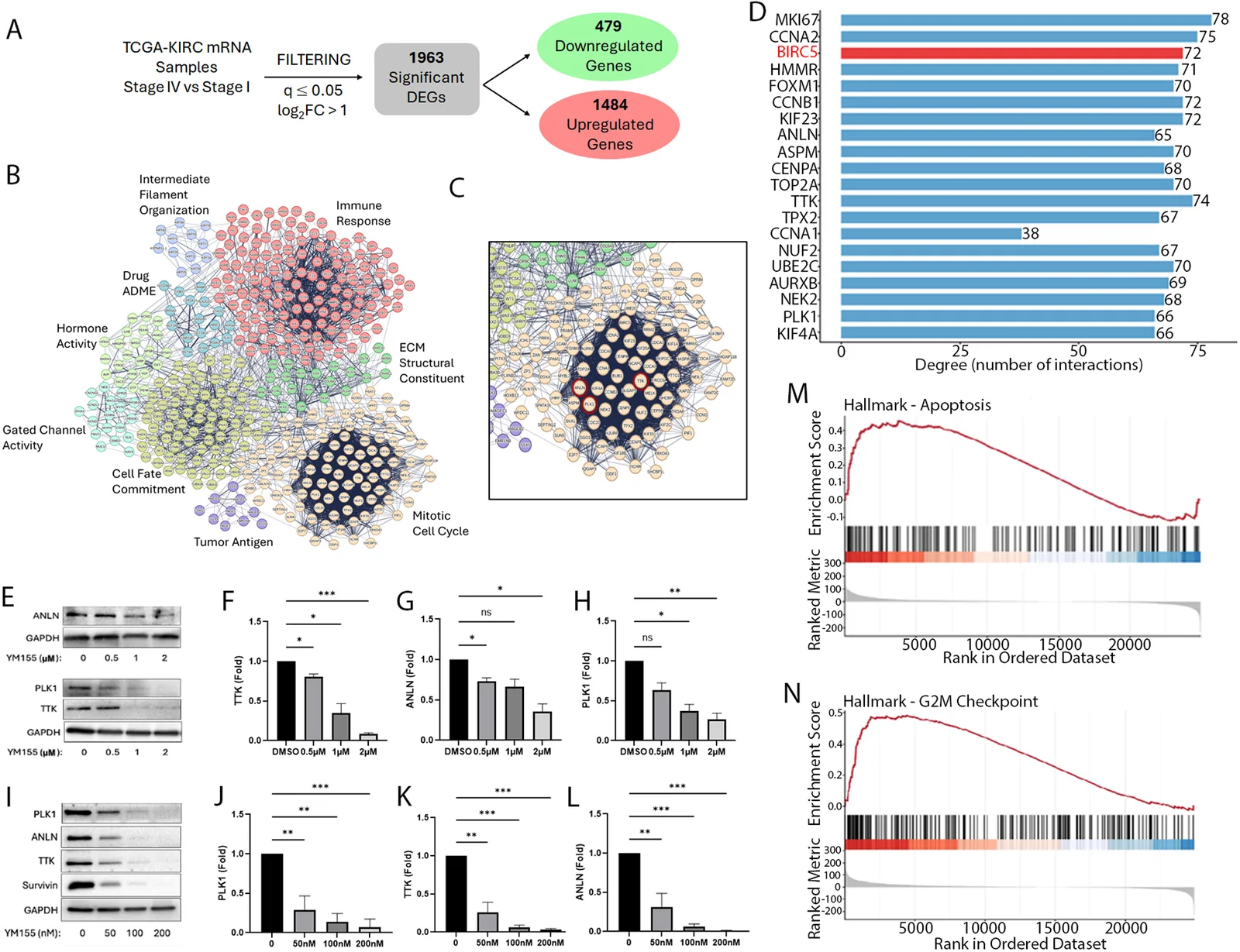
**Survivin is a central cell-cycle hub in ccRCC.** (A) Analytical workflow for differential gene expression analysis. TCGA-KIRC RNA-sequencing data (STAR counts) were compared between stage IV and stage I tumors using DESeq2 (*q*≤ 0.05, |log_2_FC|>1), yielding 1963 significant differentially expressed genes (DEGs), of which 1484 were upregulated and 479 were downregulated. (B) Protein–protein interaction (PPI) network of upregulated DEGs constructed using the STRING database (v12; combined score ≥0.4). Functional clustering reveals interconnected modules enriched for cell fate commitment, mitotic cell cycle, immune response, intermediate filament organization and extracellular matrix (ECM) structural constituents. Inset highlights the dense cell-cycle hub module containing *BIRC5*. (C) Magnified view of the mitotic cell-cycle hub module from the PPI network, showing the densely interconnected core of cell-cycle regulators. *ANLN*, *PLK1* and *TTK* (circled in red) are highlighted as representative co-expressed hub genes validated pharmacologically below. (D) Degree centrality of the top 20 cell-cycle hub genes in the STRING PPI network of significantly upregulated DEGs. *BIRC5* (red) ranks third by degree centrality, with 72 direct interaction partners, surpassed only by *MKI67* (78 direct interaction partners) and *CCNA2* (75 direct interaction partners), establishing it as one of the most highly connected cell-cycle nodes in the ccRCC interactome. (E) Representative western blots showing dose-dependent downregulation of ANLN, PLK1, TTK and survivin in RENCA cells; GAPDH served as loading control. (F-H) Quantification of TTK (F), ANLN (G) and PLK1 (H) protein levels (fold change relative to DMSO). Data represent mean±s.d.; ns, not significant; *P<0.05, **P<0.01, ***P<0.001 (one-way ANOVA with post-hoc Dunnett test); *n*=3 experiments. (I-L) Pharmacological validation in 786-O cells. Cells were treated with YM155 (0, 50, 100, 200 nM). (I) Representative western blots showing dose-dependent downregulation of PLK1, ANLN, TTK and survivin in 786-O cells; GAPDH served as loading control. Please note that the survivin and GAPDH blots shown are the same as for Fig. S1 as those targets were probed from the same lysates used here. (J-L) Quantification of TTK (J), PLK1 (K) and ANLN (L) protein levels (fold change relative to vehicle). Data represent mean±s.d.; **P<0.01, ***P<0.001 (one-way ANOVA with post-hoc Dunnett test); *n*=3 experiments. (M) Gene set enrichment analysis (GSEA) enrichment plot for HALLMARK_APOPTOSIS in TCGA-KIRC tumors versus adjacent normal tissue, showing activation of the apoptosis program in which *BIRC5* participates as a leading-edge gene. (N) GSEA enrichment plot for HALLMARK_G2M_CHECKPOINT in TCGA-KIRC tumors versus adjacent normal tissue, illustrating coordinated upregulation of the cell-cycle program in which *BIRC5* participates as a leading-edge gene.

To evaluate transcriptomic differences associated with disease progression, we analyzed publicly available TCGA-KIRC data, focusing on stage I versus stage IV tumors. After quality control and filtering, differential expression analysis identified 479 downregulated and 1484 upregulated genes (analytical workflow in Fig. 2A; DEGs summarized in Table S1). A PPI network was constructed to identify highly connected genes among the upregulated differentially expressed genes (DEGs) (Fig. 2B). Cytoscape's String application was used to filter nodes by a degree ≤2, to exclude disconnected nodes and nodes with minimal interactions. Kmeans clustering identified nine functionally distinct clusters among the upregulated. Clustering analysis revealed distinct patterns of enrichment among the upregulated DEGs, with the majority of significantly clustered genes mapping to pathways involved in cellular immune responses and cell-cycle regulation (Fig. 2B). The cluster identified for its functional role in mitotic cell cycling forms a highly interconnected gene network of key cell-cycle regulators (Fig. 2C), including survivin’s encoding gene (*BIRC5*), which ranks among the most highly connected hub genes by degree centrality (Fig. 2D). Other functionally distinct clusters in the protein–protein interaction (PPI) network included genes associated with cell fate commitment, hormonal activity, extracellular matrix structure, gated channel activity, drug metabolism, intermediate filament organization and tumor antigens. These findings suggest that advanced-stage ccRCC is characterized by marked dysregulation of cell proliferation-related processes, underscoring the central role of aberrant cell-cycle control in disease progression. To pharmacologically validate the cell-cycle hub module identified in our network analysis, we treated RENCA cells with YM155 (0, 0.5, 1, 2 μM) and examined the expression of co-regulated cell-cycle hub genes *ANLN*, *PLK1* and *TTK* by immunoblotting. YM155 treatment produced a dose-dependent reduction in all three hub proteins (Fig. 2E; quantification in Fig. 2F-H), and the same dose-dependent downregulation of PLK1, ANLN, TTK and survivin was reproduced in the human ccRCC line 786-O (YM155 50, 100, 200 nM; Fig. 2I; quantification in Fig. 2J-L). Consistent with our DEG analysis and the predicted biological consequence of disrupting this hub module, gene set enrichment analysis (GSEA) of TCGA-KIRC tumors versus adjacent normal tissue showed coordinated upregulation of cell-cycle and downregulation of apoptosis programs in which *BIRC5* participates as a leading-edge gene (HALLMARK_APOPTOSIS, Fig. 2M; HALLMARK_G2M_CHECKPOINT, Fig. 2N).

### Survivin inhibition decreases cell-cycle progression and proliferation

**Fig. 3.**
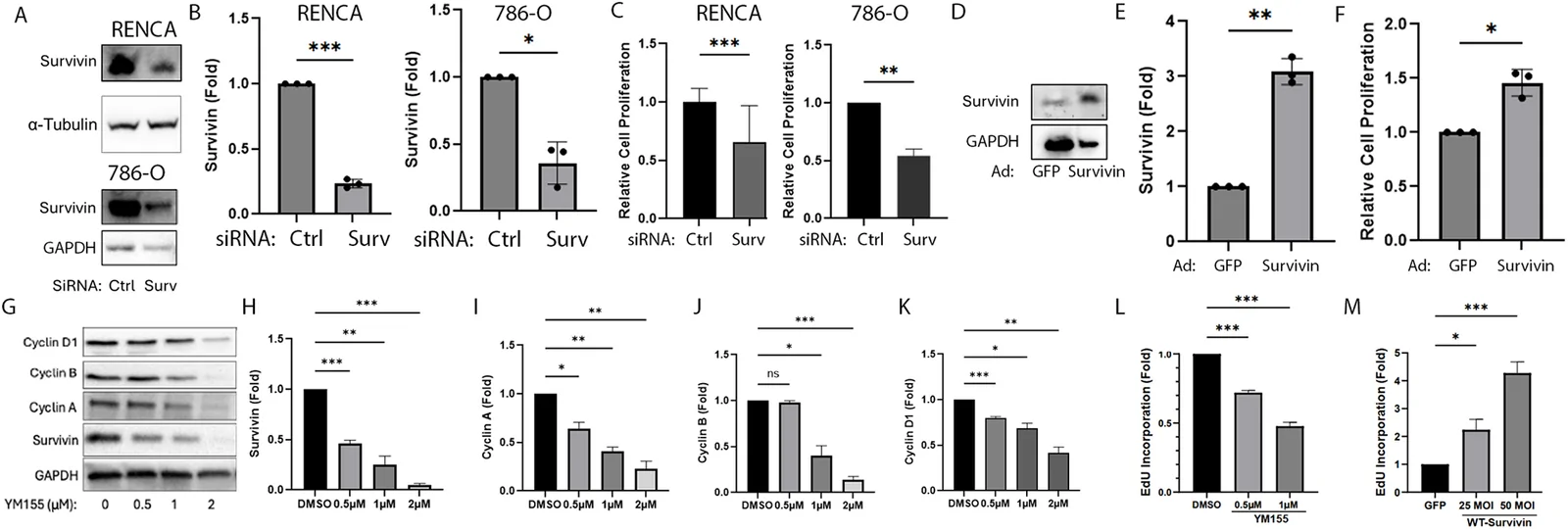
**Survivin inhibition decreases proliferation and halts cell-cycle progression.** (A) Immunoblot of survivin expression in control versus survivin siRNA-treated RENCA and 786-O cells. Tubulin or GAPDH serves as loading control, *n*=3 experiments. (B) Densitometric quantification of survivin knockdown, *n*=3 experiments; *P<0.05, ***P<0.001 (unpaired two-tailed *t*-test). (C) Bar graph depicting relative cell proliferation between control versus survivin siRNA-treated RENCA and 786-O cells. Proliferation was assessed via cell counting, *n*=3 experiments; **P<0.01, ***P<0.001 (unpaired two-tailed *t*-test). (D) Western blot of survivin in RENCA cells following adenoviral overexpression of wild-type survivin at 50 multiplicity of infection (MOI) versus control (Ad-GFP); GAPDH loading control. (E) Densitometric quantification of survivin overexpression blots for RENCA. *n*=3, mean±s.d.; **P<0.01 (unpaired two-tailed *t*-test). (F) Bar graph depicting relative cell proliferation between control versus survivin overexpression RENCA cells. Proliferation was assessed via cell counting, *n*=3 experiments; **P<0.01 (unpaired two-tailed *t*-test). (G-K) G0-synchronized RENCA cells were treated with 0.5, 1 and 2 μM YM155 for 24 h. Whole-cell lysates were collected and analyzed with immunoblotting (G) to determine protein levels of survivin (H), cyclin A (I), cyclin B (J) and cyclin D1 (K). Expression levels were normalized to α-tubulin and displayed as fold change relative to DMSO. *n*=4; ns, not significant; *P<0.05, **P<0.01, ***P<0.001 (one-way ANOVA with post-hoc Dunnett test). G0-synchronized RENCA cells were treated with 0.5, 1 and 2 μM YM155 for 24 h. (L,M) S-phase entry with YM155 treatment (L) and adenoviral overexpression (M) was assessed by 5-ethynyl-2’-deoxyuridine (EdU) incorporation. *n*=4. Data are mean±s.e.m.; *P<0.05 and ***P<0.001 (one-way ANOVA with post-hoc Dunnett test).

Previous studies have shown that survivin is a key regulator of normal and cancer cell proliferation (Jaiswal et al., 2015; Mousso et al., 2025; Biber et al., 2023; Guo et al., 2015b; Chen et al., 2016; Mobahat et al., 2014). To directly assess survivin's tumorigenic properties, we performed *in vitro* loss-of-function experiments in RENCA and human 786-O ccRCC cells using siRNA. Survivin knockdown (confirmed by western blotting, Fig. 3A for 786-O and RENCA; densitometric quantification across multiple independent replicates in Fig. 3B) caused a dramatic reduction in cancer cell proliferation compared to controls. Across replicate cell counting assays, survivin-deficient cells showed significantly lower growth rates (by ∼40% relative to control for RENCA and ∼50% relative to control for 786-O, Fig. 3C). We also overexpressed survivin using adenovirus in RENCA (Fig. 3D,E for expression and quantification, respectively) and found a ∼50% increase in cell count after survivin overexpression (Fig. 3F). To further evaluate survivin's role in RCC proliferation, we used a small-molecule inhibitor of survivin, YM155 (Guo et al., 2015b; Cheng et al., 2016; Sasaki et al., 2015). Cells were serum starved to synchronize in G0, a quiescent state, and then incubated in growth medium containing YM155 or DMSO (vehicle control) for 24 h. Immunoblotting of total cell lysates revealed that survivin inhibition significantly reduced the expression of cyclin D1, an early G1 cell-cycle regulator, together with dose-dependent reductions in survivin, cyclin A and cyclin B (Fig. 3G-K). These dose-dependent effects on survivin and cell-cycle proteins were reproduced in the human ccRCC line 786-O, in which YM155 treatment (50, 100, 200 nM) reduced survivin, cyclin A, cyclin B and cyclin D1 protein levels in a concentration-dependent manner (Fig. S1). We also found that YM155 treatment significantly reduced S-phase entry, as assessed by 5-ethynyl-2’-deoxyuridine (EdU) incorporation (Fig. 3L). To further determine whether survivin overexpression affects S-phase entry, RENCA cells were infected with a survivin adenovirus or GFP control. Survivin overexpression accelerated S-phase entry compared to that by GFP (Fig. 3M). Collectively, these findings suggest that survivin is required for cell-cycle progression and proliferation in renal carcinoma.

### Survivin inhibition decreases collective and single-cell motility

**Fig. 4.**
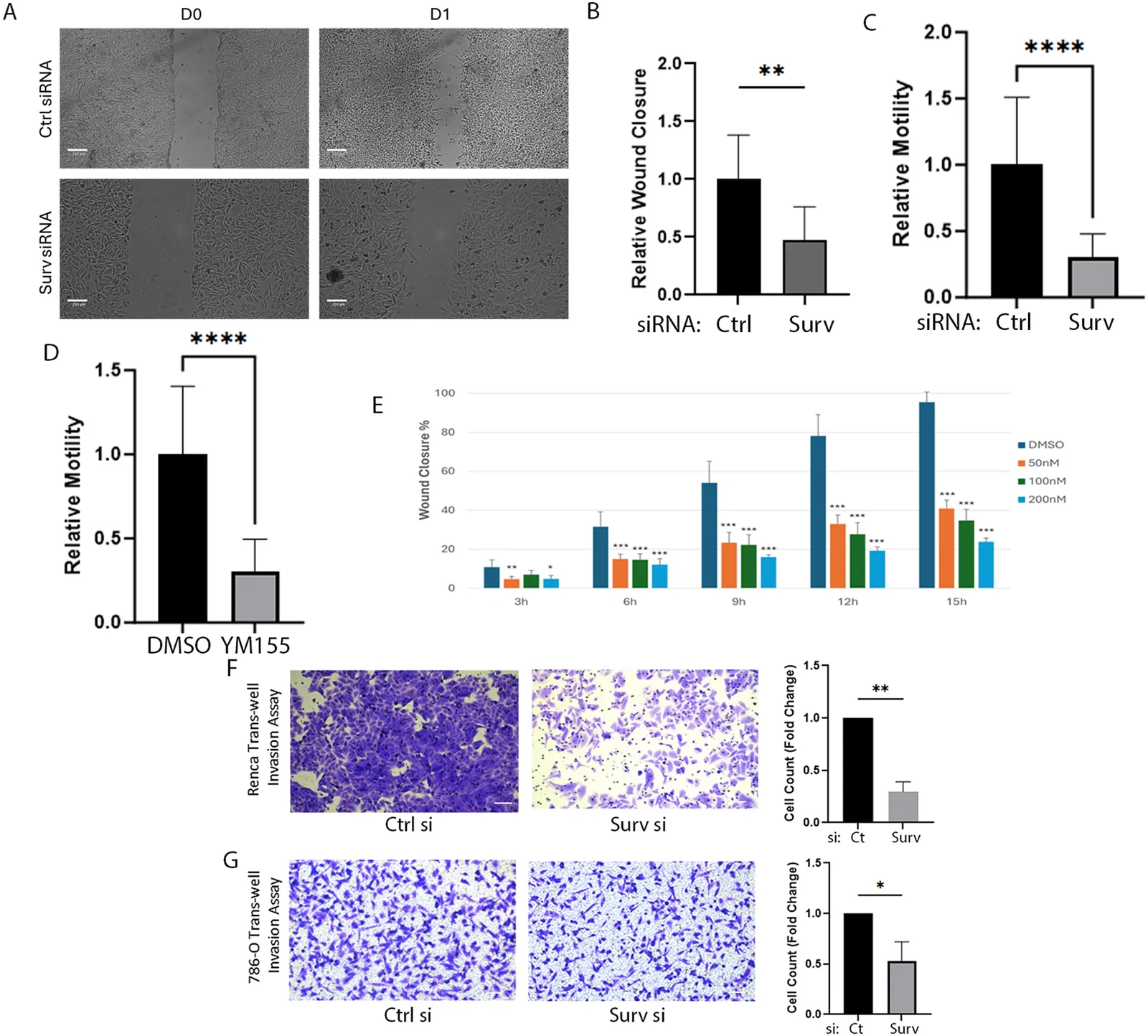
**Survivin loss impairs collective and single-cell migration in RENCA and 786-O cells.** (A) Phase-contrast images of confluent RENCA monolayers immediately [day (D)0] and 24 h (D1) after a 10-μl pipette-tip scratch under control (Ctrl) or murine *Birc5* (Surv) siRNA. Scale bars: 200 μm. (B) Relative wound closure at 24 h normalized to Ctrl siRNA. *n*=3 independent experiments, mean±s.d.; **P<0.01 (unpaired two-tailed *t*-test). (C) Relative single-cell motility (nuclear-centroid speed tracked over 120 min at 1-min intervals) of RENCA cells under Ctrl versus survivin siRNA-treated conditions, normalized to Ctrl. 60-80 cells per condition, *n*=3 independent experiments; ****P<0.0001 (unpaired two-tailed *t*-test). (D) Relative single-cell motility of RENCA cells treated with DMSO versus 1 μM YM155. 60-80 cells per condition, *n*=3 independent experiments; ****P<0.0001 (unpaired two-tailed *t*-test). (E) Time course of wound closure (%) in 786-O cells treated with DMSO or YM155 (50, 100 or 200 nM), measured at 3, 6, 9, 12 and 15 h after scratch. *n*=3 independent experiments, mean±s.d.; *P<0.05, **P<0.01, ***P<0.001 (one-way ANOVA with post-hoc Dunnett test) versus DMSO at each time point. (F,G) Representative Crystal Violet-stained transwell migration images of RENCA (F) and 786-O (G) cells transfected with scramble (left column) or *Birc5* (middle column) siRNA, with bar graph quantification (right column). *n*=3 experiments; *P<0.05, **P<0.01 (unpaired two-tailed *t*-test). Scale bars: 200 μm.

Previous studies have shown that survivin is a key regulator of normal and cancer cell migration (Nabzdyk et al., 2011; Mehrotra et al., 2010). We first performed a scratch assay by plating control or survivin siRNA-treated RENCA cells to confluency. After 24 h, survivin siRNA-treated cells closed the scratch ∼50% less than did control siRNA-treated cells, Fig. 4A,B). Because scratch assays can be complicated by proliferation effects, especially when performed over longer periods of time, we also tracked cell migration via time-lapse video single-cell migration assay. Our experimental data showed that survivin siRNA reduced migration by almost 80% (Fig. 4C) and that 1 μM YM155 treatment reduced migration by ∼70% (Fig. 4D). To address the possibility that residual proliferation contributes to wound closure, we additionally monitored scratch closure at various time points (3, 6, 9, 12 and 15 h) across a YM155 dose response in human 786-O cells (Fig. 4E). We found evidence of migration defect as early as 3 and 6 h, which is unlikely to be due to proliferation effects. As a further proliferation-independent measure, we performed Boyden-chamber transwell migration assays, which showed that survivin knockdown markedly reduced migration in both RENCA (Fig. 4F) and 786-O cells (Fig. 4G). These data suggest that reducing survivin not only stunts RCC cell proliferation but also hinders cell motility, which together would limit tumor expansion, aligning with the known pro-tumorigenic role of survivin.

### Metabolic dysfunction induced by loss of survivin

**Fig. 5.**
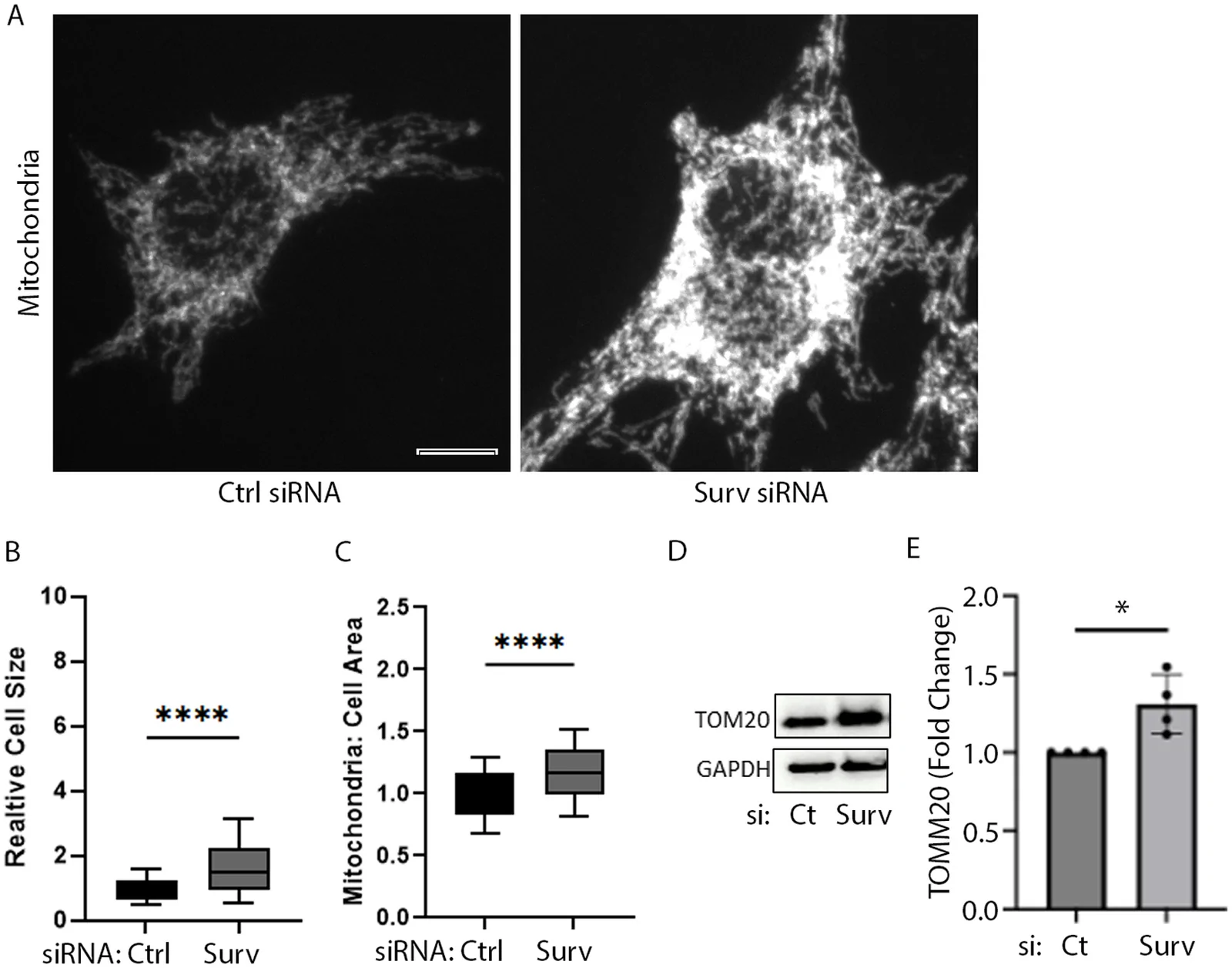
**Survivin knockdown increases cell size and mitochondria content.** (A) Representative images of RENCA cells stained with TOM20 to visualize mitochondria. Scale bar: 10 μm. (B,C) Box-and-whisker plots depicting relative cell size (B) and mitochondria/cell area (C) between control and survivin siRNA-treated RENCA cells. At least 100-120 cells were quantified from *n*=3 experiments. The bar represents the median, with the box representing the first and third quartile. Whiskers depict the tenth and 90th percentiles. ****P<0.0001 (unpaired two-tailed *t*-test). (D) Representative immunoblots of TOM20 (mitochondrial outer membrane marker) in whole-cell lysates from RENCA cells transfected with control or survivin (Surv) siRNA. GAPDH serves as a loading control. (E) Densitometric quantification of TOM20 in RENCA. *n*=4 independent experiments, mean±s.d.; *P<0.05 (unpaired two-tailed *t*-test).

In addition to functional deficits, immunostaining and image analysis demonstrated a reduction in mitochondrial content upon survivin knockdown. RENCA cells were immunostained to visualize mitochondria using a mitochondrial marker, TOM20 (also known as TOMM20), and images were captured on a widefield fluorescence microscope for quantitative analysis. Interestingly, survivin knockdown cells showed increased mitochondria content and generally larger cell morphology (Fig. 5A-C). Quantification of the cell size and mitochondrial/cell area showed increased cell size (∼50% larger) and area of mitochondria (∼20% more mitochondria) relative to those of control siRNA-treated cells. This finding was confirmed in 786-O cells as well (Fig. S2). As an orthogonal confirmation result, we also performed western blot analysis of TOM20 expression and found ∼25% increase in TOM20 expression in survivin-knockdown RENCA cells (Fig. 5D,E). To examine the function of these mitochondria, we assessed oxygen consumption rate (OCR) and found a ∼30% decrease in basal OCR (Fig. S3). To start to probe the mechanism behind how survivin may be changing mitochondria content and function, we utilized a mitophagy staining kit but did not find any obvious evidence for changes in mitophagy (Fig. S4). How survivin may be affecting mitochondria is interesting and currently a topic under investigation.

## DISCUSSION

Survivin is widely recognized for its canonical roles as an inhibitor of apoptosis and a regulated of mitotic progression, but our findings expand its functional repertoire in ccRCC. We demonstrate that survivin is not only indispensable for cell-cycle progression and proliferation but also plays a critical role in maintaining mitochondria structure, bioenergetic efficiency and cell migration. This suggests that survivin may serve as an important hub that coordinates the tumorigenesis of ccRCC. An interesting observation from our study was the increase in mitochondrial staining intensity and cell size after survivin knockdown despite a decrease in OCR. This raises the possibility of accumulation of dysfunctional mitochondria rather than enhanced biogenesis. We emphasize that the present dataset does not directly assess mitochondrial turnover, and we therefore frame impaired clearance or mitophagy as a mechanistic hypothesis to be tested. This hypothesis is informed by prior work implicating survivin in the inhibition of parkin-mediated mitophagy (Townley and Wheatley, 2020). In RCC, this disruption may promote the persistence of structurally abundant yet functionally compromised mitochondria, exacerbating bioenergetic insufficiency and sensitizing tumor cells to metabolic stress. Follow-up studies assessing mitophagy and mitochondrial turnover will be important to confirm this mechanism. We also note that YM155 has been reported to bind mitochondrial DNA directly and impair its function independently of survivin (Mondal et al., 2022). Because this off-target activity could, in principle, contribute to the mitochondrial and cellular phenotypes observed with YM155, we emphasize that our key conclusions are anchored by genetic *BIRC5* perturbation: the proliferation, cell-cycle and migration phenotypes were reproduced using two independent *BIRC5*-targeting siRNAs in different cell lines and were mirrored by survivin overexpression. The concordance between genetic knockdown and pharmacologic inhibition indicates that these effects are genuinely survivin dependent, and the YM155 data should be interpreted alongside the genetic loss- and gain-of-function results.

Our STRING PPI analysis of upregulated DEGs identifies many highly connected cell-cycle hubs, and network topology alone does not single out any one gene. Our prioritization of *BIRC5*/survivin therefore rests on the convergence of three factors. First, *BIRC5* is among the most highly connected nodes in the upregulated PPI network (degree, 72), ranking third in the top-20 cell-cycle hub list behind only *MKI67* (degree, 78) and *CCNA2* (degree, 75), placing it within the densely interconnected core of the cell-cycle/mitosis module. Second, *BIRC5* expression stratifies overall survival in TCGA-KIRC (log-rank *P*=0.00022; hazard ratio, 1.8) and is significantly elevated with increasing tumor stage and in tumor versus matched normal tissue in the GSE53757 validation cohort, providing a clinical–prognostic anchor that the higher-degree hubs (*MKI67*, *CCNA2*) provide less directly in ccRCC. Third, among the top-ranked cell-cycle hubs, *BIRC5* is the target of a clinically evaluated small molecule (YM155), enabling direct pharmacologic interrogation in both murine and human ccRCC backgrounds.

Our data also emphasize survivin's broader oncogenic functions in driving cell migration. The observation that both survivin knockdown and pharmacologic inhibition with YM155 substantially impaired RENCA cell motility suggests that survivin links proliferative and cytoskeletal programs, consistent with other findings (Guo et al., 2015a). Such dual regulation reinforces survivin's role as a facilitator of tumor expansion and metastatic potential, consistent with its strong association with advanced tumor stage and poor prognosis in patient datasets. Therapeutically, these results underscore the promise of targeting survivin in RCC. Although survivin inhibitors such as YM155 have shown preclinical efficacy, their clinical success has been limited, in part due to delivery challenges and activation of compensatory survival pathways (Albadari and Li, 2023). Our findings suggest that survivin inhibition could be more effective in rational combination regimens. For example, pairing survivin inhibitors with metabolic therapies (i.e. glycolysis or OXPHOS modulators) may exploit the mitochondrial vulnerabilities unmasked by survivin depletion. In addition, survivin's role in sustaining proliferation and motility raises the possibility of synergistic benefit when combined with cell-cycle inhibitors or immunotherapies. Survivin expression may also serve as a dual biomarker, identifying aggressive disease and predicting therapeutic response.

Functional analyses were performed in the murine RENCA and human 786-O ccRCC cell lines, and the concordance of results between species and model systems strengthens the generalizability of our findings; nonetheless, validation across additional human ccRCC lines (e.g. A498), male and female and in patient-derived xenografts will be required to strengthen translational relevance. Of note, survivin is essentially undetectable in normal human kidney epithelial cells yet is robustly expressed in RCC cell lines and tumor tissue, in which its level correlates with stage, grade and metastasis (Lei et al., 2010), and pathways that stabilize and elevate survivin promote RCC proliferation, consistent with a causal pro-tumorigenic role. Our work was also limited to *in vitro* settings, and *in vivo* studies are essential to define the systemic impact of survivin inhibition on tumor progression and host metabolism. Finally, RCC is a heterogeneous disease, and survivin's role may differ across subtypes and stages, necessitating broader investigation of context-specific functions. In summary, our findings position survivin as a multifaceted oncogenic driver in RCC that integrates cell-cycle regulation, cytoskeletal organization and mitochondrial homeostasis. Survivin depletion dismantles these interconnected networks, leading to cell-cycle arrest, impaired migration and bioenergetic collapse. By revealing survivin as a linchpin that bridges proliferative and metabolic programs, this work not only deepens our understanding of RCC biology but also highlights new opportunities for therapeutic intervention. Targeting survivin, particularly in combination with metabolic or immunomodulatory agents, represents a promising strategy to exploit the metabolic–structural vulnerabilities of RCC and improve outcomes for patients with this aggressive disease.

## MATERIALS AND METHODS

### Cell culture and siRNA transfection

RENCA cells (ATCC, CRL-2947) and the human ccRCC line 786-O (ATCC, CRL-1932) were cultured in RPMI-1640 medium supplemented with 10% fetal bovine serum (FBS), 100 U/ml penicillin and 100 μg/ml streptomycin. Cells were transfected with either survivin-specific siRNA [for RENCA, murine *Birc5* (Thermo Fisher Scientific, pooled siRNA, 160714); for 786-O, human *BIRC5* (Thermo Fisher Scientific, pooled siRNA, s1457)] or a non-targeting control siRNA using Lipofectamine RNAiMAX (Thermo Fisher Scientific, 13778075) according to the manufacturer's instructions. The siRNAs were used at a final concentration of 10 nM.

### YM-155 treatment

After 48 h of serum starvation in RPMI medium containing 1 mg/ml bovine serum albumin, RENCA cells were incubated in 10% serum-containing medium with 0.5, 1 or 2 μM YM155 (Cayman, 11490) for 24 h. For 786-O cells, 50, 100 and 200 nM YM155 was used to produce dose-dependent survivin knockdown.

### Adenovirus infection

Serum-starved RENCA cells were infected with either GFP (Vector Biolabs, 1060) or *BIRC5* (Vector Biolabs, 1611) adenoviruses at a multiplicity of infection (MOI) of 25 or 50. Cells were incubated with the adenovirus for 24 h prior to subsequent experiments.

### EdU incorporation

RENCA cells were seeded onto 12-mm glass coverslips and incubated with 20 μM EdU (Thermo Fisher Scientific, C10337) for 9 or 24 h. Following incubation, cells were fixed in 3.7% formaldehyde in PBS for 15 min. EdU incorporation assay was conducted using the Click-iT EdU Alexa Fluor 488 Imaging Kit (Thermo Fisher Scientific, C10337) according to the manufacturer's instructions. Coverslips were mounted on microscope slides using DAPI-containing mounting medium (Thermo Fisher Scientific, P36962) and sealed with nail polish. Experiments were independently repeated four times, and five fields of view per coverslip were analyzed to determine the percentage of EdU-positive RENCA cells. EdU incorporation was quantified as fold change relative to control (DMSO or GFP) as previously described.

### Cell proliferation and scratch assay

For cell proliferation assay, cells were plated per well in 24-well plates using triplicate determinations, with proliferation assessed after 72-96 h by trypsonizing and counting number of cells. For scratch assay, RENCA cells were grown to a confluent monolayer in 24-well plates, and a straight scratch was made through the cell layer using a sterile 10 μl pipette tip. After scratching, the wells were gently washed with PBS to remove debris. Wound closure was monitored by capturing images of the same scratch area at 0 and 24 h using an inverted microscope. The remaining wound area at 24 h was quantified using ImageJ software, and migration was expressed as the percentage of wound closure relative to the initial scratch area. For dose-response scratch assays, confluent RENCA or 786-O monolayers were treated with vehicle (DMSO) or YM155 (RENCA, 0.5, 1, 2 μM; 786-O, 50, 100, 200 nM), and wound closure was imaged and quantified at various timepoints to capture early, proliferation-independent migration. Cells were serum starved for 24 h prior to scratch assay to minimize proliferative effects on wound area.

### Single-cell migration assay

For single-cell migration experiments, cells were plated in 24-well plates coated with type I collagen (Millipore) overnight. Where indicated, YM-155 (Cayman, 11490) was added to the culture medium (at 1 μM concentration) overnight prior to time-lapse imaging. Time-lapse images of randomly migrating cells were collected using a 10× objective for 120 min at 1-min time intervals using cellSens (Olympus Life Science) software. The centroid of the cell nucleus was tracked using ImageJ, and the average speed of migration was computed on a per-cell basis as before (Gau et al., 2019).

### Transwell migration assay

Transwell migration was assessed using 8.0-μm pore polycarbonate inserts (uncoated). RENCA or 786-O cells transfected with scramble or survivin siRNA were seeded in the upper chamber in serum-free medium, with 10% FBS in the lower chamber as the chemoattractant. After 12 h, non-migrated cells were removed from the upper surface with a cotton swab, and migrated cells on the underside of the membrane were fixed in 4% paraformaldehyde, stained with Crystal Violet and counted in five randomly selected fields per insert.

### Mitochondrial morphology and function assessment

Mitochondrial network morphology was visualized by immunofluorescence staining of TOM20, a mitochondrial outer membrane protein. Cells were fixed with 4% paraformaldehyde for 5 min and permeabilized with 0.1% Triton X-100. Samples were incubated with a primary antibody against TOM20 (Cell Signaling Technology, D8T4N, 1:200) followed by an Alexa Fluor 647-conjugated secondary antibody (Jackson ImmunoResearch, 111-607-003, 1:100). Cells were counterstained with ActinRed555 ReadyProbes (Thermo Fisher Scientific, R37112) and DAPI. Fluorescence images were taken with either 60× objective on Olympus with Cicero confocal microscopes. All images were background subtracted using ImageJ software for quantitative analysis. Mitochondrial respiration analysis was performed using an Agilent Seahorse XF Analyzer. Briefly, RENCA cells were seeded into a XFmini eight-well cell culture microplate (Agilent) at 50,000 cells/well. Measurements were performed in a Seahorse XF Analyzer (Agilent). For the Seahorse Cell Mito Stress Test profile, cells were incubated for 1 h at 37°C in XF Seahorse medium supplemented with glucose, glutamine and sodium pyruvate and without CO_2_. OCR was measured under standard conditions and after the addition of 1 μmol/l oligomycin, 1.5 μmol/l FCCP and 1 μmol/l rotenone/1 μmol/l Antimycin A.

### Gene expression analysis

RNA-sequencing data and associated clinical annotations for KIRC were obtained from TCGA. Only patient data annotated with stage I or stage IV were included in the analysis; patients with intermediate stages (II-III) were excluded. Raw count data from stage I and stage IV tumors were normalized, and the DESeq2 package in R was used to identify DEGs between the tumor stages. DEGs were defined as those with a log_2_foldchange>1 and a Benjamini–Hochberg adjusted *P*-value (FDR)<0.05 (Love et al., 2014).

### Clustering analysis

Cytoscape's String application was used to create a network of PPIs for the upregulated DEGs (Szklarczyk et al., 2015). Nodes (DEGs) with a degree of 2 or less were excluded to minimize small, disconnected clusters. The resulting network was clustered using kmeans clustering, with nine designated clusters, to create distinct regions of gene interactions. The clusters were named by their most enriched Gene Ontology, and the network was displayed on the STRING online server for visualization purposes.

### Protein extraction and immunoblotting

Total cell lysates were collected as follows. RENCA cells were washed twice with cold 1× DPBS and then incubated with 5× sample buffer [250 mM Tris-HCl (pH 6.8), 10% sodium dodecyl sulfate, 50% glycerol, 0.02% Bromophenol Blue and 1 0mM 2-mercaptoethanol] for 2 min. Cells were then scraped ten times using a cell scraper. Protein samples were denatured at 100°C, and equal amounts were fractionated on 6-15% SDS-PAGE gels, then transferred electrophoretically onto polyvinylidene difluoride membranes using Bio-Rad's Trans-Blot Turbo Transfer System. Membranes were blocked in 5% milk in 1× Tris-buffered saline with 0.1% Tween 20 (TBST) prior to antibody incubation. Primary antibodies against survivin (Novus Biologicals, NB500-201; 1:500), cyclin D1 (Santa Cruz Biotechnology, sc-20044; 1:200), cyclin B (BioLegend, 647902, 1:500), cyclin A (Santa Cruz Biotechnology, sc-239, 1:200), anillin (Thermo Fisher Scientific, PA5-114851, 1:500), TTK (Thermo Fisher Scientific, 35-9100, 1:500), PLK1 (Santa Cruz Biotechnology, sc-17783, 1:500), TOM20 (Thermo Fisher Scientific, MA5-34964, 1:1000), GAPDH (loading control; Protein Tech, 10494-1-AP, 1:5000) and tubulin (loading control; Sigma-Aldrich, T5168, 1:1000) were incubated at room temperature for 1 h and overnight at 4°C. Following incubation, membranes were washed three times with 1× TBST and then probed with secondary antibodies (HRP-conjugated Affinipure Goat Anti-Rabbit IgG, Proteintech, SA00001-2 or HRP-conjugated Affinipure Goat Anti-Mouse IgG, Proteintech, SA00001-1) for 1 h at room temperature. Membranes were washed three times with 1× TBST prior to imaging. Antibody signals were detected using Clarity Western ECL Substrate (Bio-Rad, 170-5061) or Clarity Max Western ECL Substrate (Bio-Rad, 1705062). All image analyses were performed using Bio-Rad Image Lab Software. Protein levels of survivin were normalized to GAPDH or tubulin as a loading control, and protein levels were displayed as a fold change.

### Statistical analysis

All experiments were conducted with sufficient biological replicates (*n*=3 or 4) for each condition. Data are presented as mean±s.d., unless otherwise stated. Statistical comparisons between two groups (e.g. control versus survivin knockdown) were made using unpaired two-tailed Student's *t*-tests. Comparisons between multiple groups were made by a one-way ANOVA with a Dunnett correction for multiple comparisons. *P*<0.05 was considered statistically significant for all analyses. GraphPad Prism (v8.0) was used for data plotting and statistical calculations.

## Supplementary Material

10.1242/dmm.052706_sup1 Supplementary information

Table S1. Up and downregulated DEGs from TCGA analysis of KIRC dataset between stage I and stage IV cancers.
