# Supplementary material for: Survivin drives renal cell carcinoma tumorigenesis via proliferative and metabolic adaptation
Source: Dis Model Mech. 2026 Aug 25;19(8):dmm052706. doi: 10.1242/dmm.052706 (PMC13580557; doi:10.1242/dmm.052706)
Supplement: Supplementary file 1 [file dmm-19-052706-s1.pdf]

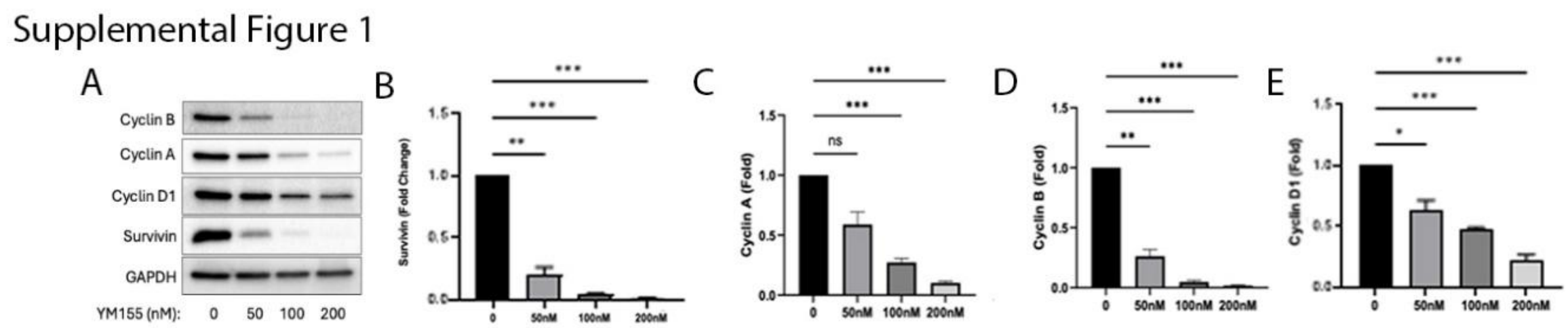

Fig. S1. Survivin loss affects cell cycle progression in 786-O cells. A) Western blot of cyclin B, cyclin A, cyclin D1, survivin and GAPDH (loading control). B-E) Densitometric quantification of survivin (B), cyclin A (C), cyclin B (D) and cyclin D1 (E), expressed as fold change vs vehicle (ns, not significant; *P<0.05, **P<0.01, ***P<0.001). Please note that the survivin and GAPDH blots are the same as those in Fig. 2I as those targets were probed from the same lysates used here.

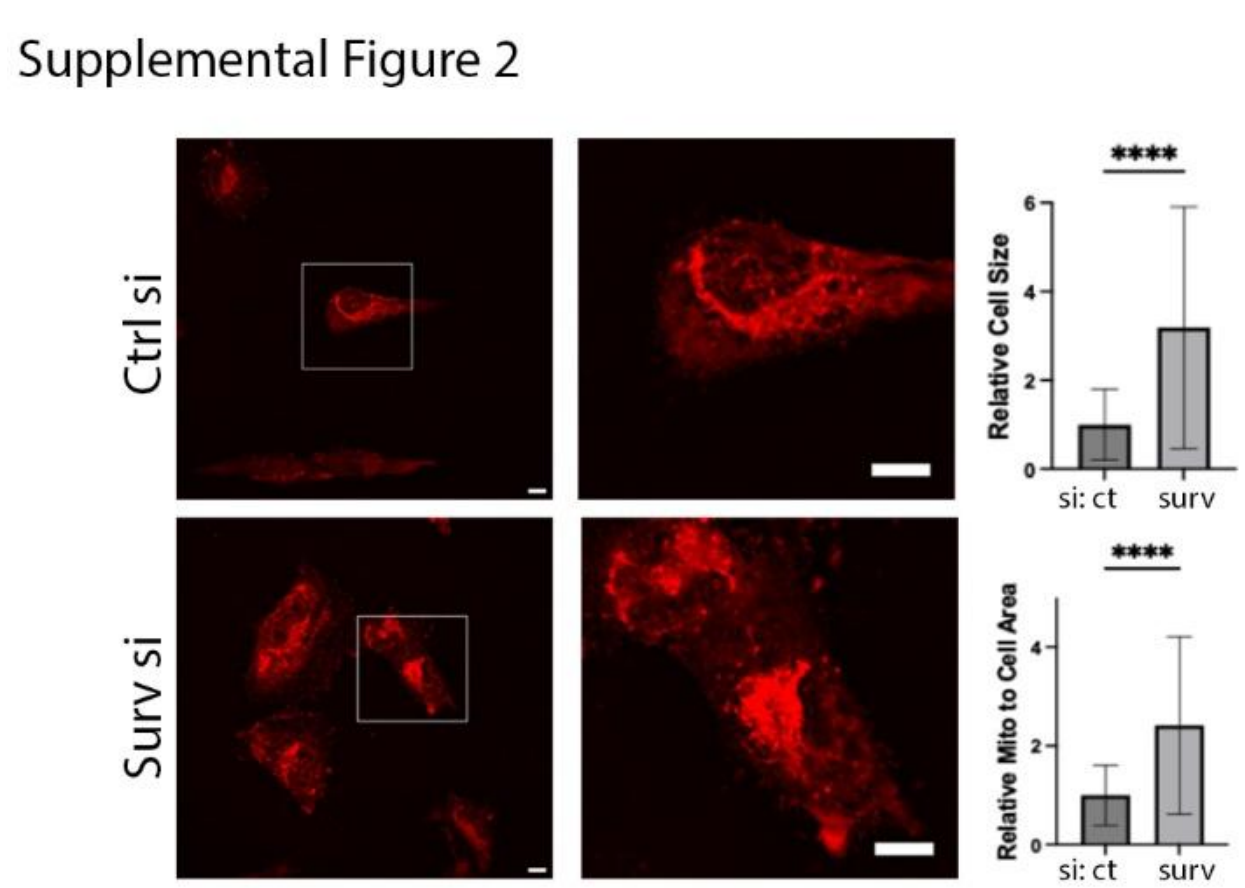

Fig. S2. Survivin regulates 786-O cell size and mitochondria content. Representative images of mitochondria-stained 786-O cells with ctrl or surv siRNA treatment, with bar graph quantification of cell size and mito/cell area. n=3 experiments, ****P<0.0001.

Disease Models & Mechanisms • Supplementary information

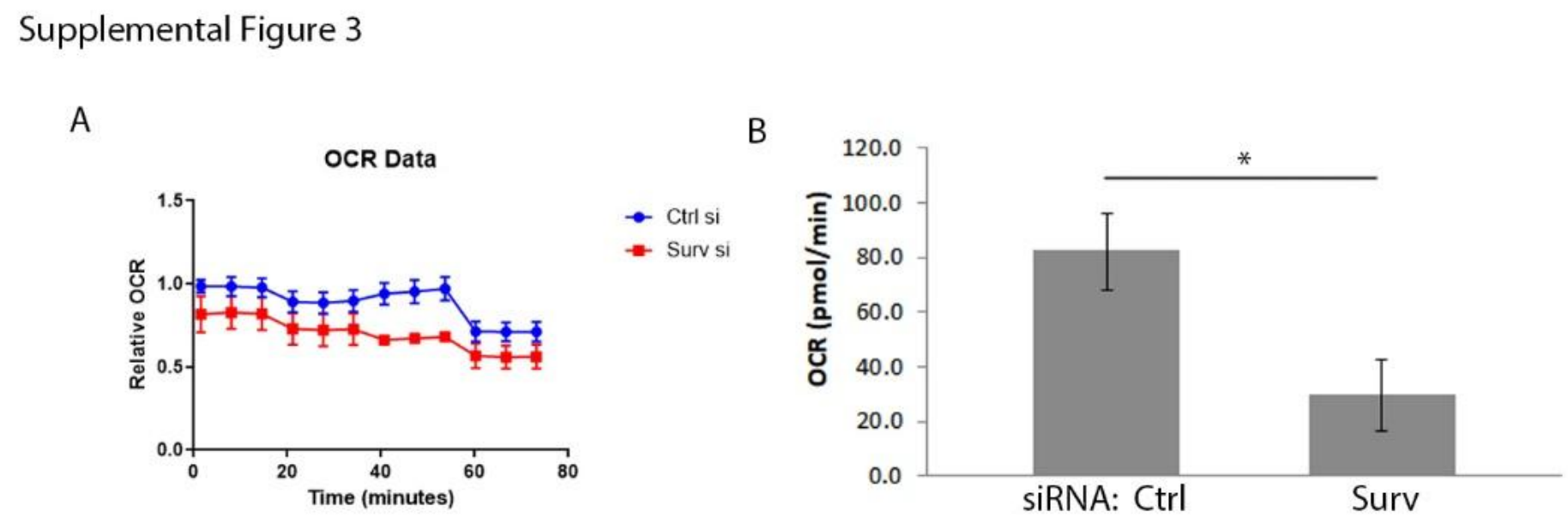

Fig. S3. Loss of survivin reduces RENCA cell oxygen consumption rate. A) Representative OCR curve from ctrl vs surv siRNA RENCA cells. B) Bar graph showing OCR (pmol/min) of cells treated with survivin siRNA. *n*=3 experiments. **P*<0.05.

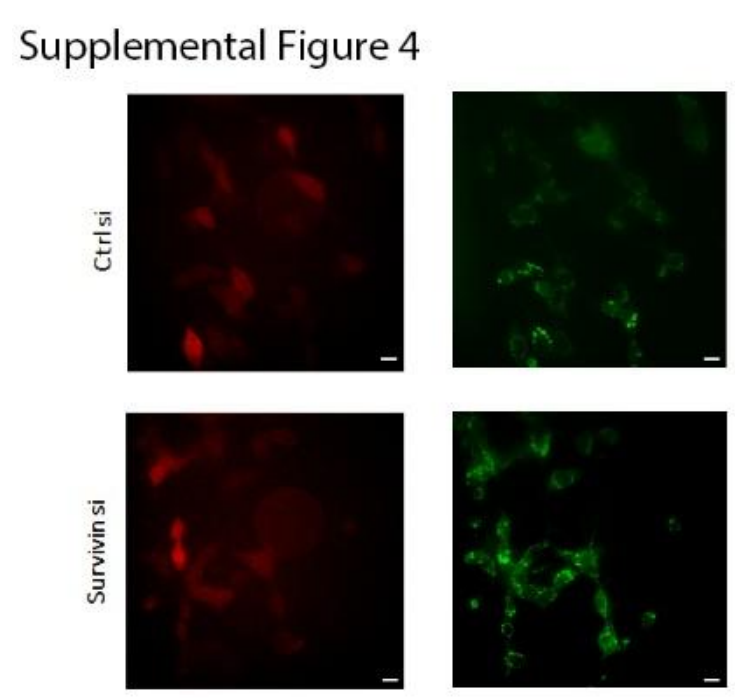

Fig. S4. Survivin knockdown does not affect mitophagy. RENCA cells were transfected with non targeting ctrl siRNA (top) or survivin targeting siRNA (bottom), labeled with the Mitophagy Detection Kit (MD01, Dojindo), and imaged by confocal microscopy. Red: Mtphagy Dye (555 nm excitation), which accumulates in mitochondria and fluoresces strongly when they fuse with acidic lysosomes, reporting mitophagy. Green: Lyso Dye (470 nm excitation), marking lysosomes. Co localization of the two signals indicates delivery of mitochondria to lysosomes. Images are representative [single optical sections/maximum intensity projections of an 8 plane *z* stack acquired at 0.26 µm steps]. Scale bar: 10µm.

Table S1. Up and downregulated DEGs from TCGA analysis of KIRC dataset between stage I and stage IV cancers.

Available for download at
https://journals.biologists.com/dmm/article-lookup/doi/10.1242/dmm.052706#supplementary-data

Disease Models & Mechanisms • Supplementary information
